# Glutamatergic System in Depression and Its Role in Neuromodulatory Techniques Optimization

**DOI:** 10.3389/fpsyt.2022.886918

**Published:** 2022-04-14

**Authors:** Mohamed Adil Shah Khoodoruth, Maria Anayali Estudillo-Guerra, Kevin Pacheco-Barrios, Azan Nyundo, Gina Chapa-Koloffon, Sami Ouanes

**Affiliations:** ^1^Department of Psychiatry, Hamad Medical Corporation, Doha, Qatar; ^2^Department of Physical Medicine and Rehabilitation, Harvard Medical School, Spaulding Rehabilitation Hospital, Boston, MA, United States; ^3^Neuromodulation Center and Center for Clinical Research Learning, Harvard Medical School, Spaulding Rehabilitation Hospital and Massachusetts General Hospital, Boston, MA, United States; ^4^Universidad San Ignacio de Loyola, Vicerrectorado de Investigación, Unidad de Investigación para la Generación y Síntesis de Evidencias en Salud, Lima, Peru; ^5^Department of Psychiatry and Mental Health, School of Medicine and Dental Health, The University of Dodoma, Dodoma, Tanzania; ^6^Hospital Infantil de México Federico Gómez, Mexico City, Mexico

**Keywords:** glutamate, neuromodulation, TMS (repetitive transcranial magnetic stimulation), direct current stimulation (tDCS), photo-biomodulation therapy, treatment-resistant depression, depression, mini review

## Abstract

Depressive disorders are among the most common psychiatric conditions and contribute to significant morbidity. Even though the use of antidepressants revolutionized the management of depression and had a tremendous positive impact on the patient's outcome, a significant proportion of patients with major depressive disorder (MDD) show no or partial or response even with adequate treatment. Given the limitations of the prevailing monoamine hypothesis-based pharmacotherapy, glutamate and glutamatergic related pathways may offer an alternative and a complementary option for designing novel intervention strategies. Over the past few decades, there has been a growing interest in understanding the neurobiological underpinnings of glutamatergic dysfunctions in the pathogenesis of depressive disorders and the development of new pharmacological and non-pharmacological treatment options. There is a growing body of evidence for the efficacy of neuromodulation techniques, including transcranial magnetic stimulation, transcutaneous direct current stimulation, transcranial alternating current stimulation, and photo-biomodulation on improving connectivity and neuroplasticity associated with depression. This review attempts to revisit the role of glutamatergic neurotransmission in the etiopathogenesis of depressive disorders and review the current neuroimaging, neurophysiological and clinical evidence of these neuromodulation techniques in the pathophysiology and treatment of depression.

## Introduction

Different theories were put forward to explain the etiopathogenesis of depression. This has been a major challenge for the development of treatment options, since only 30% of patients with MDD who receive an adequate treatment experience full remission (1). Some of the neurobiological factors theoretically associated with depression are the activation of the inflammatory system, hypothalamic-pituitary-adrenal axis disturbances, dysfunctional neuroanatomic circuits (particularly the default mode network), abnormal neural activity, neurotransmitter dysfunction, polymorphisms in the 5-HTT promoter region (5HTTLPR) and interactions between brain derived neurotrophic factor (BDNF); and neurotrophic tyrosine kinase receptor 2 (NTRK2) polymorphisms (2).

Theories of neurotransmitter dysfunction in depression are well established. The monoamine theory proposes that depression is caused by a decrease of the extracellular availability of noradrenaline and serotonin. Hence, the therapeutic effect of antidepressants aimed to increase their availability. Despite the multiple studies about various multimodal pharmacological and psychological treatments of depression, the evidence for the efficacy and tolerability of these treatment options remains insufficient (3).

The delayed, and in many cases poor, response to the current antidepressants may suggest that there are more neurotransmitters involved in the pathophysiology of depression than those accounted for by the monoamine theory. Prominent among them is the glutamatergic system which may contribute to the dysfunction and perceived treatment poor response (2). Given the complexity of mood disorders and the setbacks of previous monoaminergic targets, there has been growing evidence about non-invasive neuromodulating techniques that target networks such as the limbic-cortical system framework, producing both local and regional effects (4). Growing evidence from animal and human studies shows that structural pathology, network, and connectivity dysfunction related to deficits in excitatory glutamate neurons and inhibitory GABA interneurons in the cortical and limbic areas of the brain are implicated in the genesis of depressive symptoms. As current pharmacotherapeutic treatment for depression which focused on the monoamine hypothesis has significant limitations, glutamatergic related mechanisms promise to produce superior therapeutic interventions (5). This article reviews the proposed glutamatergic mechanisms of neuromodulation in the treatment of depression.

## Glutamatergic System and Depression

**Figure 1:**
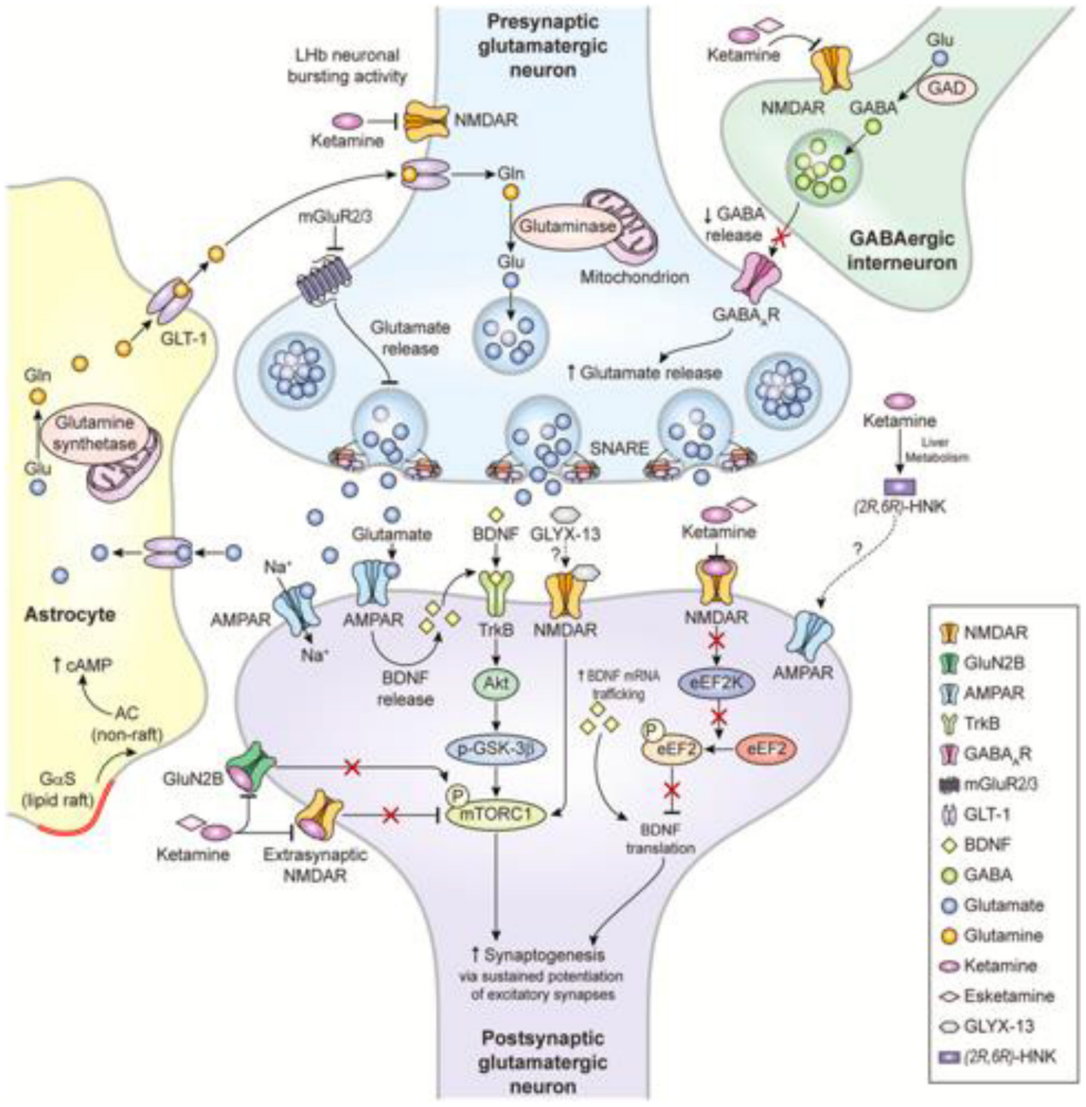
Proposed mechanisms of action of glutamatergic modulators and other putative rapid-acting antidepressants (10).

Glutamate is the principal excitatory neurotransmitter in the brain, and glutamatergic mechanisms play significant roles in nearly all key functions affected in depressed states (6). Postmortem studies have put forward evidence linking glial cell abnormalities, whose role is synaptic glutamate removal, and the pathophysiology of mood disorders (7). Lower glutamine/glutamate levels have also been found in the cortex of patients with depression (8). The “much faster than usual” antidepressant effect of ketamine, discussed later, further solidifies the role of glutamate in treatment-resistant major depression. Ionotropic (N-methyl-D-aspartate [NMDA], α-Amino-3-Hydroxy-5-Methylisoxazole-4-Propionic Acid Receptors [AMPA], and kainate receptors [not discussed herein] and metabotropic (mGluR) glutamate receptors have been shown to be responsible for modulation of mood and associated functions that are impaired in depression (9). These observations have brought about the hypothesis that NMDA and/or AMPA receptors might be novel therapeutic targets for treating depression. Figure 1 summarizes the interplay among NMDA-R inhibition, gamma aminobutyric acid (GABA)-ergic interneuron disinhibition, and ketamine metabolites hydroxynorketamines (HNK), taking ketamine—a glutamatergic agent that has been intensively investigated over the past 20 years—as an example.

### Ionotropic Receptors

These receptors are all cation-permeable, homo- and heteromeric blocks of structurally distinct subunits (11). Even though all three classes of ionotropic receptor pertain modulatory (allosteric) sites, the NMDA receptor (NMDA-R), a tetrameric heteromeric assembly of NR1 and NR2 subunits which contains glycine and glutamate recognition sites, respectively, is unique in possessing a co-agonist site and an additional site accessible to open channel blockers such as ketamine, memantine, and amantadine.

In several animal models, NMDA-R antagonists have been shown to produce antidepressant-like effects (12, 13). In humans, Crane (14, 15) was the first to report beneficial effects of high-dose D-cycloserine (DCS), an agent with NMDA-R activity initially developed to treat tuberculosis, on depressed mood, insomnia and anorexia. Although the antidepressant effects of NMDA-R blockers have not been consistent, a potent augmentation of antidepressant activity has been hypothesized when ketamine, an NMDA antagonist, was co-administered with other antidepressants to patients with depression (16, 17). NMDA antagonists are also not devoid of psychotomimetic, motor, and other adverse side effects, that might be mitigated by the use of CP-101,606, an NR2B-subunit antagonist (9).

Historically, NMDARs have received more attention as they are key players in the process of excitotoxicity, a pivotal mechanism in stroke and neurodegenerative disorders. While NMDA receptors allow entry of Ca^2+^, AMPA receptors heterotetramer, which consists of four glutamate receptor subunits (GluR1-4), mediate the entry of Na^+^ current into the neuron, thus resulting in rapid signal transmission (18). Compared with NMDA receptors in depression, AMPA receptors seem to be understimulated (19). Positive allosteric modulator Org 26576 has shown greater symptomatic improvement compared with placebo, as well as good tolerability and pharmacokinetic properties (20). Similarly, previous studies on drugs that combine serotonin uptake inhibition with positive allosteric modulation of AMPA receptors have shown that AMPA receptors potentiators (ARPs) or “AMPAkines” may augment the activity and perhaps expedite the onset of the therapeutic effects of biogenic amine and second messenger-based antidepressants (18, 21).

### Ketamine: One Drug, Many Uses

Originally approved in 1970 as an anesthetic, ketamine thereafter earned notoriety as a recreational drug of abuse (“Special K”) because of its dissociative effects. Subsequently, in 2019, the Food and Drug Administration (FDA) approved the S-enantiomer of ketamine, esketamine, for patients with treatment-resistant depression. Although many monoaminergic treatments exist, at least a third of patients do not have a response after two or more trials of antidepressant drugs and are considered to have treatment-resistant depression (22). When evidence emerged about its rapid (within several hours) antidepressant effects (23), ketamine revolutionized the landscape as there was desperate need for urgent relief of suicidal crises and faster restoration of functioning, thereby providing a respite on economic and social burdens of treatment-resistant depression. Efficacy and safety studies (Studies 3002 and 3003) of esketamine in treatment-resistant depression showed its impressive and rapid onset of action on day 2 of treatment, along with its efficacy beyond 1 month in patients who had an initial response (24).

Ketamine is a mixture of two enantiomers (mirror image molecules): R- and S-ketamine. (2S,6S)- and (2R,6R)-HNK are ketamine's major metabolites (10). Esketamine's product monograph enumerates increased blood pressure, dissociation, dizziness, and nausea among others as common side effects (25). Nonetheless, Zanos et al. (26) indicated that (2R,6R)-HNK did not induce any of the side effects typically associated with ketamine.

Besides, some clinical trials have shown that by modulating rather than inhibiting NMDARs, may prevent ketamine's NMDAR inhibition-mediated side effects. For example, partial agonists at the NMDAR glycine B binding site, such as GLYX-13 (rapastinel) and D-cycloserine, produced antidepressant effects in both human and animal studies (27). Furthermore, another likely more potent and novel NMDAR enhancer, sarcosine, a glycine transporter-1 (GlyT-1) inhibitor, showed improvement in depressive behaviors and symptoms (28, 29). Another novel NMDAR enhancer which looks promising in the treatment of mood disorders is sodium benzoate, a D-amino acid oxidase (DAAO) inhibitor which prevents degradation of D-amino acid (30). A recent clinical trial demonstrated that sodium benzoate can decrease perceived stress, improve cognitive function, and enhance treatment adherence in late-life depression (31).

### Metabotropic Receptors

Metabotropic glutamatergic receptors (mGluR)[homodimers], which are mainly distributed in corticolimbic areas, can be broadly divided into three categories according to their structures, ligand recognition profiles and coupling to cellular transduction systems: group I (mGluR1 and 5) receptors, group II (mGluR2 and 3), and group III (mGluR4, 6,7 and 8) (32).

Group I mGluR antagonists mimic certain properties of NMDA antagonists to some extent such as their influence on mood, as well as their neuroprotective effects (32, 33). This similarity could be explained by the indirect facilitation of NMDA receptors by presynaptic group I receptors, and thus promoting glutamate release in cortico-limbic areas such as the amygdala (34). Moreover, it has been shown that mGluR 5 antagonists have antidepressant actions in animal models of acute and chronic stress (35).

While group I receptors are positively coupled with Phospholipase C *via* Gq, group II and III receptors, on the other hand, are positively coupled to Gi, leading to an inhibitory effect on adenylyl cyclase (32, 36) which subsequently suppresses the release of glutamate (37, 38) and in turn modulates depressive states. As shown in Figure 1, mGluR 2/3 antagonists are thought to enhance synaptic glutamate levels, thereby boosting AMPAR transmission and firing rates and extracellular monoamine levels. A few articles have suggested that the antidepressant effects of group II antagonists is linked to their induction of glutamate release in the serotonin-rich dorsal raphe nucleus (DRN) (39, 40). The DRN has been strongly associated with mood disorders such as depression (41). Furthermore, group III agonists have been found to possess antidepressant effects in rodent models by reducing excessive glutamate release (42, 43).

## Assessing the Glutamatergic System

The assessment of glutamate and glutamate receptors is of high interest across many neuropsychiatric disorders. However, compared to other neurotransmitter systems, its *in-vivo* assessments pose several challenges (44). The most common assessment approach is the so-called “molecular imaging” with positron emission tomography (PET) and single photon emission computed tomography (SPECT). These techniques have the advantage of being an *in vivo* technique and having an excellent safety profile (45). Specific tracers are available for PET/SPECT molecular imaging of subtypes of the ionotropic (NMDA, AMPA, and kainate receptors) and metabotropic glutamate receptors (mGluRs).

Due to conformational issues and the difficulty of utilizing brain-penetrating ligands for amino acid receptors, there has been little advance in developing radiotracers for ionotropic glutamate receptors, with the exception of the NMDA receptor (GluN2B subunit ligand). On the other hand, mGluR imaging, particularly for subtypes 5 and 1, has been found effective (44), and has been used in studying the pathophysiology, endophenotypes, and drug development for depression (46–48).

Moreover, ^1^H magnetic resonance spectroscopy (MRS) is also a non-invasive method for the assessment of levels of neurometabolites including glutamatergic ones in the brain (49). MRS takes advantage of differential protons' magnetic fields depending on their chemical environment. Using this property, it helps distinguish between different chemical compounds, and quantify their concentrations, including glutamate-related metabolites (50). The metabolites are measured in a specified volume, the voxel. MRS was widely used in depression studies showing that patients with depression had lower glutamate and glutathione (GSH) levels; however, due to overlapping signals and low concentrations, this method is prone to signal-to-noise ratio challenges. New methodological improvements are needed to boost this ratio and increase the likelihood of generating robust and reliable results in the study of depression (51, 52).

Furthermore, one can indirectly assess the glutamatergic synapsis in the cortex using transcranial magnetic stimulation (TMS) with paired pulse protocol. The intracortical facilitation (ICF) metric has been associated with glutamate tonus in the motor cortex (53, 54). In particular, pharmacological studies with paired-pulse TMS have demonstrated that both GABA-A agonist (55, 56) and NMDA antagonist reduced ICF (57, 58). Thus, ICF is assumed to be mainly associated with glutamate receptor-mediated excitatory functions in the motor cortex. Only few studies have assessed the role of ICF in depression with heterogenous results (59–61). New evidence on glutamatergic tonus from non-motor cortex (such as DLPFC) areas with combined TMS-EEG protocols is needed.

Although, none of the above methods is the perfect and reliable technique to assess glutamatergic pathways in patients with depression. The combination of clinical and multimodal neuroimaging assessments is the best approach for robust and reproducible protocols.

## Modulatory Techniques That Target Glutamate Pathways

The International Neuromodulation Society defines therapeutic neuromodulation as “the alteration of nerve activity through targeted delivery of a stimulus, such as electrical stimulation or chemical agents, to specific neurological sites in the body (62).

In the last decades, several non-invasive brain stimulation techniques have been used to treat depression in humans, including rTMS or tDCS. Reduced connectivity in the prefrontal cortex and anterior cingulate gyrus and reduced neuroplasticity are found in depressive disorders and play important roles in the pathophysiology of depression (63–66).

Brain stimulation's effect on depression might be through neuroplasticity modulation. Electroconvulsive therapy (ECT) is among the oldest brain stimulation techniques that have been used to treat refractory depression. ECT impacts plasticity-associated transcripts and their proteins in the hippocampus (67). Also, several clinical studies have shown an increase in connectivity and cerebellar volume in patients with MDD (68–72) and white matter modulation in the pathways between the frontal and limbic areas after receiving ECT therapy (73). Animal models showed that ECT influences the glutamatergic system; rat models with decreased glutamate, exhibited NR2B upregulation after ECT.

TMS is a non-invasive technique of brain stimulation that uses the principles of electromagnetic induction to produce an electrical current in the brain surface. A magnetic field induces a secondary electrical current. There are different types of TMS including rTMS which generates repetitive magnetic pulses from seconds to minutes. rTMS is a Food and Drug Administration (FDA) approved treatment for patients with MDD who are resistant to antidepressant treatment. rTMS might stimulate neurogenesis like ECT, and also modulate brain activity and neurotransmitters including dopamine and serotonin. Cortical excitability modulation might be impaired in patients with depression, and TMS might improve cortical modulation (74), and modulate functional connectivity between the central executive work and default mode network (75).

### Transcutaneous Direct Current Stimulation TDCS

tDCS is another noninvasive brain stimulation technique for the treatment of depressive disorders. The device of tDCS has two electrodes, anode, and cathode. It applies a constant low current (0.5–2mA) *via* electrodes on the scalp and changes the cortical excitability.

At a neuronal level, tDCS modulates the resting membrane potential in a polarity-dependent fashion: anodal stimulation increases cortical excitability in the stimulated region while cathodal decreases it.

A recent meta-analysis that included 27 randomized controlled trials concluded that tDCS was effective in the treatment of depression when compared to sham (76). Also, some authors consider that tDCS should be considered as “definitely effective” (Level A) given the current evidence.

### Transcranial Alternating Current Stimulation (TACS)

Another stimulation approach used to modulate endogenous brain activity is tACS, which applies a weak bidirectional and biphasic current with a sine-wave pattern to the scalp for a duration of 2–5 min (lower than the typical time for stimulation for tDCS which is 20 min) (77). Three recent articles have showcased the potential therapeutic effects of tACS for the treatment of MDD and cognitive impairment: one well-structured double-blind randomized clinical trial (78) and two case reports (79, 80). In a nutshell, these three papers seem to indicate that tACS is a safe, even during pregnancy, and efficacious long-term tool in MDD treatment research. Proposed mechanisms of action for tACS have been changes in cortical excitability, in brain electrical activity, and biochemical changes including neurotransmitter release (77). This non-invasive brain stimulation paradigm deserves further investigation in larger randomized trials and with various neurophysiologic assessments, especially that the mechanism of tACS, in association with the glutamatergic pathway, has been understudied.

### Photo-Biomodulation

Light has a wide range of effects on physiological and behavioral functions, including circadian rhythm, mood, and cognition (81, 82).

Recent research studies support antidepressant effects of light therapy, while light deprivation can induce depressive-like behaviors, which further indicates that light signals are a powerful modulator of mood-related behaviors.

Photo-biomodulation therapy (PBMT) is a novel and non-invasive therapy based on delivering photons in the range of red to near-infrared (NIR) spectra (600–1,100 nm) inside tissues (83, 84). PBMT can efficiently penetrate biological tissues including the CNS and produce beneficial photo-biomodulation effects; some of the proposed mechanisms of action are the increase in ATP synthesis, neurogenesis stimulation, increase on brain perfusion, and decrease of inflammation (85).

Recently, several animal and clinical studies on MDD have shown that PBMT can induce antidepressant-like effects when the prefrontal cortex (PFC) is targeted (86, 87). However, the mechanism by which PBMT ameliorates glutamatergic dysfunction to display the antidepressant phenotype is unclear.

Zhan et al. found in an animal model, that tPBMT decreased extracellular glutamate levels *via* upregulation of glutamate transporter-1 (GLT-1), alleviated dendritic atrophy and upregulated the expression of AMPA receptors on the postsynaptic membrane. This was associated with behaviorally significant antidepressant effects in mice exposed to chronic unpredictable mild stress (88).

### Vagal Nerve Stimulation (VNS)

VNS, initially developed for seizure disorders, came into the spotlight when patients reported improvements in mood after being treated for epilepsy. VNS involves surgically implanting a vagal nerve stimulator in the upper left area of the chest wall which then provides electrical stimulation to the vagal (tenth cranial) nerve. The stimulation lasts for 30 s, and can be turned on or off by the patient by holding a magnet over the device. The largest VNS study targeting treatment-resistant MDD (failure to respond to medications and ECT) consisted of 235 patients who received either VNS or a placebo/sham treatment (89). The duration of treatment was over 12 weeks and there was only a 15% positive response rate and 10% remission rate. However, positive response increased to 34% over time (90). This finding suggests that VNS would not be recommended for an acute debilitating depressive episode or high-risk clinical situations of harm to self or others. Nonetheless, this treatment is worth considering as an adjunct to medication management. One theory behind the antidepressant effects of VNS is the decrease of glutamate concentration in the rostral anterior cingulate cortex (91).

## Current Neurophysiological and Clinical Evidence

Non-invasive brain stimulation offers a potential alternative, non-pharmacologic approach to treat unipolar depression and treatment resistant depression (TRD), even though there is some evidence about the efficacy and safety of non-invasive neuromodulation techniques to treat unipolar depression, especially in the case of ECT and rTMS (92).

The rationale for neuromodulation for depression is based on a neural network theory that posits a specific set of structurally and functionally connected brain regions that work together to maintain normal mood regulation (93, 94), however, little is known about the specific mechanisms.

The putative cellular mechanisms of action to this group of therapies involve the action over the glutamatergic neuronal firing, depending on the technique and polarity used, TMS and ECT will depolarize the cell membrane and generate an action potential while tDCS have an impact on the action potential depending on the polarity, cathodal stimulation will decrease, and anodal stimulation will increase the action potential. The after-effects are mediated by “long-term potentiation (LTP)-like” mechanisms where the upregulation of neurotransmitter release facilitates the opening of AMPARs and indirectly that of NMDARs (95). The mechanism of action of tDCS in psychiatric disorders has been attributed to the effects of anodal stimulation (96). Studies often report the anode placement over the frontal cortex while the cathodal electrode placement reports are more inconsistent, however, there are reports of inflammation reduction and neuroprotective effects by cathodal stimulation (97).

In this review, we looked for the possible clinical impact of different neuromodulation techniques over glutamate pathways and we found three rTMS studies (Table 1): 2 with TRD patients (98, 99) and another with MDD patients (100) that were not taking any pharmacological treatment. MRI spectroscopy was performed before and after the intervention to assess the glutamate/GABA ratio (Table 1). The studies found modifications on the glutamatergic and GABAergic neurotransmission after high-frequency rTMS treatment on DLPFC. Specifically, an increase of the glutamine/creatine ratio (100) and GABA concentrations (98, 99) on the left DLPFC after ~20–25 daily sessions. Indeed, this justify further research on glutamate biomarkers for the optimization of rTMS protocols in depression.

**Table 1:** Evidence on glutamate modulation by drugs and by tDCS.

**Author**	**Subjects**	**Intervention /Study design**	**Outcomes**	**Results**
**Evidence on glutamate modulation by drugs**				
Chris Baeken et al. (98)	Eighteen antidepressant-free unipolar treatment-resistant depressed (TRD) patient.	Sham-controlled accelerated high frequency (aHF)-rTMS	MR spectroscopy study applied to the left dorsolateral prefrontal cortex (DLPFC). Neurochemical concentrations in the bilateral DLPFC and rostral anterior cingulate cortex (rACC).	– Compared to healthy individuals, TRD patients displayed significantly lower baseline glutamate and glutamine concentrations in the left DLPFC. – aHF-rTMS does not significantly alter neurochemical concentrations in the three predefined brain regions. – Clinical improvement was related to significant GABA concentration increases in the left DLPFC.
Dubin et al. 2016 (99)	Twenty-three with TRD (7 men)	A 5-week naturalistic, open-label treatment study of rTMS, 10-Hz rTMS over the left dorsolateral prefrontal cortex (DLPFC) rTMS pulses over the left DLPFC at 10 Hz and 80%−120% of motor threshold for 25 daily sessions, with each session consisting of pulses	Hamilton Rating Scale for Depression (HAMD24). – Levels of medial prefrontal cortex (MPFC) γ-aminobutyric acid (GABA) and the combined resonance of glutamate and glutamine (Glx) as assessed *in vivo* with proton magnetic resonance spectroscopy (1H MRS).	– GABA in the MPFC increased 13.8% (*p =* 0.013) in all depressed individuals after treatment. –No significant effects of rTMS on Glx. – GABA and Glx were highly correlated in severely depressed patients at baseline but not after TMS.
Erbay MF et al. (100)	Eighteen patients with MMD without pharmacological treatment (10 female, 8 male)	20 sessions of rTMS directed at the left DLPFC over a 2-week period	The Hamilton Depression Scale (HAMD) H magnetic resonance spectroscopy (H-MRS)	–Statistically significant differences in HAMD scores before and after rTMS. (*p < * 0.001) –The peak metabolite ratios of NAA/Cr, GSH/Cr, and Gln/Cr were significantly higher after rTMS compared to those before rTMS. (*p =* 0.016, *p =* 0.040 and *p =* 0.008, respectively)
**Evidence on glutamate modulation by tDCS**				
Filmer et al. (101)	47 healthy subjects	tDCS at left PFC Across three sessions, subjects received anodal, cathodal, or sham stimulation. In the case of anodal and cathodal sessions, the stimulation was applied for 9 minutes including 30-s ramp up and down, at an intensity of 0.7 mA	Behavioral tasks performance: –visual search, go no go. – ravens (intelligence) – psychological refractory period (PRP) paradigm. MRS GABA and glutamate the ratio of GABA to glutamate.	–Cathodal stimulation reduced training-related reaction times [BF10 = 3.446, t(46) = 2.16, p = 0.018]. GABA and glutamate in the prefrontal cortex were associated with the disrupted response selection training. –Subjects who showed a higher level of inhibition (more GABA relative to glutamate) in the prefrontal cortex were affected by stimulation to a greater extent, showing higher levels of disruption to response selection training gains in active tDCS sessions compared to sham.
Mezger E et al. (102)	20 healthy subjects, 12= women	Double-blind cross-over design, 20 subjects were randomized to active tDCS with standard bifrontal montage, anode over the left dorsolateral prefrontal cortex (DLPFC) and the cathode over the right DLPFC. Two combined tDCS-MRI sessions (approximately 2 h each)	–PANAS trait and state questionnaire (Positive And Negative Affect Schedule) –(MRS) to quantify glutamate (Glu), Glu + glutamine (Glx) and gamma aminobutyric acid (GABA) concentration. –Resting-state functional connectivity MRI (rsfcMRI)	For both conditions PANAS scores were higher before (meanactive = 16.00 = 9.6; mean sham = 14.95 = 7.1) compared to after (mean active = 13.25 = 8; mean sham= 12.15 = 8.6) the stimulation. There were no significant changes of Glu, Glx and GABA levels across conditions. Female subjects showed a significant reduction in Glu concentration in the active compared to the sham condition (β = 0.03 [0.01–0.05], t(140) = 2.87, *p =* 0.004, *d* = 1.29 [0.41–2.17]), while male subjects did not. After active tDCS, there was an increase within thesubgenual/subcallosal cortex (at trend level; clustercorrected at 20 voxels, cluster: *x* = 2; *y* = 28; *z* = – 22 (21 voxel); log-*p* value = 1.0; FDR-corrected.

Given that deficits in glutamate in cortical and limbic regions are implicated in MDD and causally related to depressive-like behaviors; treatment that reverses or normalizes glutamate can improve depressive symptoms. Glutamate receptors, specifically the metabotropic subgroup II receptors of mGluRs are targets of interest in developing novel antidepressants (103). Preclinical studies have demonstrated that the antagonism and negative allosteric modulation of mGlu2/3 receptor has antidepressant properties (104, 105). Indeed, the expression mGluR2/3 receptors reduction is observed in the anterior cingulate cortex among patients with MDD (106, 107). Furthermore, significant increase in CSF levels of glutamate and glutamine among MDD patients compared to healthy control (108) has been observed and linked to hyperactivation of NMDA receptors (109), inflammatory process (110, 111) and serotonergic signaling (111). Similarly, a systematic review and meta-analysis evidence shows that even peripheral blood glutamate levels were significantly increased in MDD patients compared to the controls (112).

While pharmacological techniques have targeted the glutamatergic receptors as a therapeutic target for treating depressive disorders, neuromodulation techniques offer alternative or augmentative treatment for the same. Non-invasive brain stimulation techniques including the non-convulsive TMS and tDCS and convulsive ECT and MST provide safe options targeting specific areas in dorsolateral prefrontal cortex (DLPFC) which is a key site for the frontoparietal network (FPN). In hypoactive state, (FPN) is associated with hyperactivity of the default mode network (DMN) that may promote depressive behavior and cognitions such as self-referential processing, depressive rumination and negative bias observed in MDD (113–115).

Provided at high frequency (HF-TMS), low frequency (LF-TMS), theta-bursts (TBS) and deep (dTMS) (116, 117); clinical trials have demonstrated the efficacy and safety of TMS treatment. HF-TMS over the left DLPFC is shown to have a superior effectiveness compared to sham treatment in individuals with depression even when used without concomitant antidepressant (118, 119), and has the benefit of accelerating clinical response (120) and also effective as a monotherapy for both unipolar or bipolar depression (121). In case of poor response to HF-rTMS, LF-TMS can be especially advantageous and safe when there is high risk of seizures and poor tolerability to pain (117, 122).

Similar to TMS techniques, tDCS targets left DLPFC primarily aiming to counterbalance the hyperactivity of the DMN secondary to hypoactivity of the frontoparietal network of the left DLPFC (123). tDCS combined with antidepressant produce faster and greater response compared to the antidepressant alone or tDCS alone (119). In a non-inferiority trial, tDCS failed to show its non-inferiority to escitalopram, but was still superior to placebo (124).

As for convulsive modalities, ECT has been used for decades as second line treatment for MDD and in some cases such as acute suicidal or psychotic features, it can be used as a first line. ECT is now considered safe, effective and tolerable with increased efficacy with increased in association with medications, when combined with nortriptyline and lithium and venlafaxine and lithium (125, 126). MST is a TMS variant with a more focused superficial field and minimal stimulation of inner brain structures such as hippocampus (127), thus having lower incidences of cognitive deficits unlike ECT (128) but with antidepressant effect similar to RUL ECT (129, 130). Animal models reveal a better understanding of the antidepressant mechanism of neuromodulatory techniques such as deep brain stimulation (DBS), which is observed to work through activating brain derived neurotrophic factor (BDNF) related pathways. DBS targeting the ventral medial prefrontal cortex is thought to exert its antidepressant-like and cognitive enhancement effect by increasing expression of BDNF, Akt, and mammalian target of rapamycin (mTOR), and observed to restore stress-induced synaptic loss in the hippocampus (131).

While abnormal projection of excitatory glutamate neurons in the brain regions could contribute to structural alteration, emotional stress and depression alter GABAergic function that impairs the fine-tune and control glutamate related excitatory function leading to dysfunction in these neural circuits (132, 133). Brain imaging studies have shown structural and functional alterations in depression, particularly the decreased volume of the hippocampus and the subgenual and anterior cingulate cortex of the prefrontal cortex (134, 135). Functional brain imaging shows dysfunction in three major networks related to depression, including default mode network (DMN) responsible for resting taste introspection and ruminations, the salience network (SAL) processing salient information from external sources and the central network (CEN) responsible for working memory and attention (136). Unlike the monoamine system with the main role of providing extrinsic inputs to the cerebral cortex, Glutamate provides both excitatory and inhibitory control of information flow in the brain (132, 137, 138).

The alteration of glutamate in CSF, blood and brain tissue related to synthesis, metabolism and reuptake into neuronal or glial cells is linked to decreased glutamate in the subgenual of anterior cingulate cortex associated with reduced connectivity with insula and decreased BOLD response to emotional stimuli in MDD patients (139). Alteration of the glutamatergic pathway could be linked to acute and chronic stress affecting specific circuits relevant to increased or decreased connectivity observed in individuals with MDD. Indeed, chronic stress is thought to cause atrophy of glutamate neurons contributing in volume reduction in cortical and limbic structures implicated in depression (63, 140). The alteration of glutamatergic neurons secondary to stress could also be attributed to neuroendocrine mechanism related to HPA and elevation of adrenal glucocorticoids which are implicated in the etiopathogenesis of depression. While administration of glucocorticoids may lead to dendritic atrophy and reduction in number of synapsis in the hippocampus and PFC, survival of neurons may be enhanced by downstream regulation of glucocorticoids by major neurotrophic factors such as (BDNF) which plays a role in regulation and function of neurons (141–145).

## Conclusion

Non-invasive brain stimulation offers a potential alternative, non-pharmacological approach to treat unipolar depression and TRD. While pharmacological techniques have targeted the glutamatergic receptors as a therapeutic target for treating depressive disorders, neuromodulation techniques offer alternative or augmentative treatment for the same. Non-invasive brain stimulation techniques including the non-convulsive TMS and tDCS and convulsive ECT and MST provide safe options targeting specific areas in DLPFC which is a key site for the frontoparietal network (FPN). The rationale for neuromodulation for depression is based on a neural network theory that posits a specific set of structurally and functionally connected brain regions that work together to maintain normal mood regulation. However, little is known about the specific mechanisms and further research is needed to investigate the considerable potential of ionotropic and metabotropic receptors in relation to multi-target treatment of depressive disorders.

## Author Contributions

ME-G and KP-B conceptualized the idea. MK coordinated research activity planning and execution. GC-K acquired funding and worked on references. MK and SO reviewed and edited the manuscript. All authors approved the final version of the manuscript. All authors participated in writing the first draft of the manuscript.

## Funding

The publication of this article was funded by Children's Hospital of Mexico Federico Gomez.

## Conflict of Interest

The authors declare that the research was conducted in the absence of any commercial or financial relationships that could be construed as a potential conflict of interest.

## Publisher's Note

All claims expressed in this article are solely those of the authors and do not necessarily represent those of their affiliated organizations, or those of the publisher, the editors and the reviewers. Any product that may be evaluated in this article, or claim that may be made by its manufacturer, is not guaranteed or endorsed by the publisher.
